# Incidence of Duchenne muscular dystrophy in the modern era; an Australian study

**DOI:** 10.1038/s41431-022-01138-2

**Published:** 2022-06-27

**Authors:** Didu Kariyawasam, Arlene D’Silva, David Mowat, Jacqui Russell, Hugo Sampaio, Kristi Jones, Peter Taylor, Michelle Farrar

**Affiliations:** 1 Department of Neurology, Sydney Children’s Hospital, Randwick, Sydney, NSW Australia; 2 School of Clinical Medicine, UNSW Medicine and Health, Randwick Clinical Campus, Discipline of Paediatrics, University of New South Wales, Sydney, NSW Australia; 3 Centre for Clinical Genetics, Sydney Children’s Hospital, Randwick, Sydney, NSW Australia; 4 Department of Clinical Genetics, Children’s Hospital Westmead, Westmead, NSW Australia; 5 Paediatrics and Child Health, Sydney Medical School, University of Sydney NSW Australia, Sydney, NSW Australia; 6 Genomic Diagnostics, Healius Pathology, Melbourne, Vic Australia

**Keywords:** Epidemiology, Paediatrics

## Abstract

Duchenne muscular dystrophy (DMD), an X-linked recessive condition is maternally inherited in two-thirds of affected boys. It is important to establish carrier status of female relatives to restore reproductive confidence for non-carriers and facilitate reproductive options and cardiac surveillance for carriers. This study investigates disease incidence within an Australian model of cascade screening and evolving genetic diagnostic technologies. A retrospective population-based cohort study of all genetically and/or histopathologically confirmed males with DMD, born in New South Wales and the Australian Capital Territory was undertaken from 2002–2012. Cases were identified using state-wide molecular laboratory and clinical databases. The annual disease incidence and “theoretically” preventable cases were extrapolated over the study period. Proband genotype/phenotype, pedigree analysis, carrier-risk and extent of cascade screening were also determined. The cumulative incidence of disease was 19.7 per 100,000 male live births and 1 in 5076 live born males were diagnosed with DMD. Differences in disease incidence were not statistically different when compared between 2002–2007 and 2008–2012 (incidence rate ratio = 1.13, 95% CI 0.76–1.69, *p* = 0.52). The incidence rate ratio of theoretically preventable cases did not significantly change between 2002–2007 and 2008–2012 (incidence rate ratio = 2.07, 95% CI 0.58–9.21, *p* = 0.23). Current diagnostic and cascade screening models have limitations in their impact on disease incidence, due to a spectrum of logistical, patient and condition related factors. Innovative approaches to reduce DMD incidence may be better achieved by preconception or early pregnancy carrier screening, prenatal exome sequencing and newborn screening.

## Introduction

Duchenne muscular dystrophy (DMD), an X-linked recessive condition, is the commonest form of muscular dystrophy with a reported global incidence of 1 in 3500 to 1 in 5000 live male births [1, 2]. Affected children usually present with gait disturbance including gross motor delay and/or functional motor decline by the age of 5 years [3], becoming wheelchair dependant by on average 13 years of age [4]. Despite symptomatic management with corticosteroids, proactive surveillance and intervention of comorbidities, mean survival is limited mainly by cardiorespiratory complications, to 29 years of age (for males born after 1970) [5].

DMD is caused by single or multiple exonic deletions/duplications in the dystrophin gene in 80% of cases [6]. These variants usually disrupt the translational reading frame of the gene, leading to absence of functional dystrophin protein. Point variants (nonsense or missense), small deletions, small duplications, insertions and structural rearrangements are less common causes of DMD.

The causative variants are maternally inherited in approximately two-thirds of affected boys, with de novo DMD variants arising in the remaining one-third of cases [7]. First-degree female relatives of a known carrier each have up to 50% chance of being a carrier. Carriers may remain asymptomatic or manifest a spectrum of symptoms from mild to severe muscle cramps or weakness. They also have a higher incidence of complications including dilated cardiomyopathy, leading to overt cardiac failure in 10% of cases necessitating medical intervention and in rare cases, cardiac transplantation [8]. Thus, ascertaining their carrier status is vital for personal health surveillance. In non-carrier mothers with an affected son, gonadal mosaicism confers a residual chance of recurrence of 10–15% for an another affected male or carrier daughter in each subsequent pregnancy [9, 10].

While the clinical landscape encompasses advanced therapeutic approaches in development that provide hope, corticosteroids are currently the only disease modifying therapy adopted as standard of care. Recently five other variant-specific, high-cost antisense oligonucleotides have received accelerated or conditional approval by the United States Food and Drug Administration or the European Medicines Agency, however there is an ongoing high unmet need and burden of disease such that a model of care that informs reproductive choice continues to be a central pillar in DMD families.

Prior to the discovery of the dystrophin gene and protein product in 1986, [11] diagnostic confirmation of DMD was based on a protocol of clinical examination, family history, raised creatine kinase (CK) and open muscle biopsy [12]. The advent of multiplex polymerase chain reaction in the early 1990’s enabled common deletions to be identified [13]. Other types of pathogenic variants including duplications, point variants (nonsense, missense and splicing) and structural rearrangements were not detected by this method. Determination of carrier status remained complex and was based on the triad of pedigree, CK and linkage analysis. This lack of certainty led to terminations of male pregnancies, a pathway which was formerly considered a viable reproductive option [14]. Foetal muscle biopsies from the early 1990’s onwards became an option in some centres to improve post-conception reproductive decision making for those with undetermined or high risk carrier status [15]. Quantitative methods such as Multiple Ligation Probe Assay (MLPA) enabled detection of up to 70% of children with DMD gene deletions/duplications from the early 2000’s, augmented with the diagnostic capabilities of high-throughput methods such as next generation sequencing (NGS), identifying small variants and confirming diagnosis in over 90% of affected cases [13]. Combined variant detection protocols involving MLPA and DNA sequencing had significant advantages over conventional methods for the determination of carrier state, with at least an additional 33% of female relatives of affected individuals having their carrier status clarified [16].

In conjunction with formalised genetic counselling for consenting relatives of DMD patients, genetic screening may be offered to at-risk relatives of an index case in a systematic and sequential manner, based on the probability that they will have a positive screen for the identified variant. This process is known as ‘active cascade screening’ [17]. Accordingly, clinical neurogenetics services in New South Wales (NSW), Australia have offered a centralised clinical and molecular diagnostic service and cascade screening and counselling for families of symptomatic DMD boys since 1975.

Across international jurisdictions, DMD cascade screening has been variable, leading to a significant proportion of potential carriers being unaware of their reproductive status and personal health risks [18, 19]. A study in the Netherlands noted that only 34% of potential female DMD carriers at 50% risk had been seen or offered testing [20]. Consequently, there is variability on the impact of cascade screening for DMD with some (particularly older epidemiological) studies showing stable disease incidence despite proactive cascade screening and counselling [21]. A state-specific Australian incidence study in the 1970’s concluded that cascade screening alone was insufficient to reduce incidence rates significantly [22].

Internationally, only three epidemiological studies spanning Europe and USA have been conducted from 2002 onwards for DMD, [21, 23–25] with no recent Australasian specific studies pertaining to the last decade [22, 26]. Thus, the recent impact on DMD disease incidence has not been fully elucidated since the availability of comprehensive molecular genetic diagnosis and in the context of increasing opportunity to facilitate reproductive choices for affected families through pre-implantation genetic diagnosis and prenatal genetic testing [27, 28]. This evidence gap has been highlighted as a foundation from which to extrapolate the healthcare burden and economic impact of DMD, especially due to the cumulatively progressive nature of the disease associated with a high requirement for multidisciplinary care [29].

As the paradigm shifts towards individualising and improving the timing and quality of reproductive choices for prospective parents, [30] it has become imperative to address this evidence gap and understand DMD disease incidence within current health infrastructure. Ascertaining current disease incidence will elucidate whether cascade screening goes far enough to inform reproductive choice, facilitates the interpretation of the impact of new models of genetic screening and the utility of novel diagnostic tools and serves as a foundation on which to understand the epidemiological effects of emerging disease modifying treatments and improved standards of care in DMD [19].

### Aims

1. The primary aim of the study was to establish the incidence of DMD within the state of New South Wales (NSW) and the Australian Capital Territory (ACT), Australia over the period of 2002–2012.
2. The secondary aims of the study were to investigate the extent and uptake of genetic (cascade) screening and counselling across the pedigree of affected individuals (known as probands) and to determine if this active cascade screening model, the advent of new molecular genomic technologies to identify genetic status (particularly MLPA and DNA sequencing) and access to reproductive technology, correlated with changes in disease incidence and the number of theoretically preventable cases.

## Materials and methods

This was a retrospective population-based cohort study of all genetically and/or histopathologically (through western blot and immunohistochemistry detection of dystrophin levels in muscle biopsies) confirmed males with DMD in NSW and ACT identified from 2002–2012. The ACT lies within the geographical boundaries of NSW and children from this region have traditionally had their clinical management provided by the NSW Health Service.

It is generally accepted that most boys with DMD will be diagnosed by 6 years of age [19], therefore male births for the period from 2013 onwards were not included in the study. Children with DMD born prior to 2002 have been reported previously [31]. Participants in our study included all live born males with DMD.

To reduce ascertainment bias, probands were identified using two electronic (clinical and molecular diagnostic laboratory) databases. In NSW, genetic identification of DMD in the paediatric population was undertaken by the NSW Health Pathology laboratory in the majority of cases. Probands with out of frame variants in the dystrophin gene (including those with single or multiple exonic deletions/duplications and point insertions/deletions) and those with in-frame variants but with a clinical evolution consistent with DMD and/or muscle biopsy showing absence of dystrophin, were included from this database.

A second state-wide clinical genetics database was accessed to identify probands seen by clinical geneticists over the study interval. By using a filter with defined search terms of ‘Duchenne’, ‘neuromuscular’ and ‘dystrophinopathy’ clinical cases were identified.

The date and place of birth were determined for each potential proband and those born in NSW/ACT were included. Children with a birth date on and after 1st January 2002 and on or before 31st December 2012 were included for analysis.

For all probands a medical record review was undertaken, and demographic and clinical data systematically extracted from these records. The latter included age and clinical features of initial presentation and age at diagnosis. Variant type and genetic methodology used for genotyping were also ascertained. During the study interval, genomic methodologies for dystrophin analysis included targeted gene deletion/duplication analysis, multiplex ligation-dependent probe amplification (MLPA) and DNA sequencing of the dystrophin gene.

Pedigree data was taken from genetic records and the presence of a family history of neuromuscular disease or DMD in the same generation and/or the preceding generation(s) was identified. Particularly the number of “theoretically” preventable cases was also ascertained (defined as a proband with a male with confirmed DMD in previous generations and/or a male with DMD, above the age of 6 years in the same sibship or generation).

From the pedigree data, potential carriers were identified and divided into three categories based on their relationship to the proband: first degree (sister/mother), second degree (aunts/grandmother) and third degree (or more distant) relative. Potential carriers under 16 years at the time of analysis were excluded as carrier status was not always determined by this age. Information was collated about the number of potential carriers offered genetic counselling and the number who subsequently accessed genetic testing.

### Statistical analysis

Clinical and genetic data from the study was analysed using descriptive statistics, utilising frequency and percentage measures. The annual disease incidence rate was defined as the number of new cases of DMD seen in each year of the study, compared to the number of live male births in NSW/ACT in that year. The number of theoretically preventable cases (probands with a family history in previous generation or older sibling with DMD aged >6 years) were denoted over four study intervals and compared to the number of live births to obtain incidence of theoretically preventable cases. Birth rate statistics were sourced from the Australian Bureau of Statistics, a Department of the Australian Federal Government. The annual birth rate was classified as those for the combined male live births (MLB) of NSW and ACT. The incidence rate ratio of total and theoretically preventable cases was determined by comparing incidence rates over the first six years (2002–2007) and last five years (2008–2012) of the study period, to encompass time frames when quantitative genomic methodologies were beginning to be introduced (former) and a period where combined variant analysis techniques were in keeping with current standards of best practice (latter). The confidence interval of the incidence rate ratio uses exact Poisson method, with the p value obtained using Chi^2^-statistic.

## Results

A total of 107 males were born with DMD in NSW/ACT over the study period. Probands presented at a mean age of 4.8 years (standard deviation 2.2 years), after first parental concerns at a mean of 1.8 years (standard deviation 1.1 years), and in the majority of cases (61/107; 57%) secondary to signs of gross motor delay.

The annual incidence of DMD ranged between 9.3 in 100,000 MLB in 2011 to 31.9 in 100,000 MLB in 2009 (Table 1).

**Table 1:** Duchenne muscular dystrophy annual disease incidence from 2002–2012 in New South Wales and the Australian Capital Territory.

Year of birth	Total number of DMD cases	Combined male live births NSW/ACT	Incidence of DMD per 100,000 MLB
2002	10	46,451	21.5
2003	10	46,659	21.4
2004	10	46,495	21.5
2005	9	46,669	19.2
2006	10	47,398	21.0
2007	10	48,346	20.7
2008	5	50,790	9.8
2009	16	50,161	31.9
2010	9	51,792	17.4
2011	5	53,694	9.3
2012	13	53,444	24.3
**Total**	**107**	**541,899**	**19.7**

*DMD* Duchenne muscular dystrophy, *MLB* male live births.

The cumulative incidence of disease was 19.7 per 100,000 MLB, thus 1 in 5076 live born males were diagnosed with DMD during this period. Although annual incidence fluctuated over the study interval, differences in disease incidence in NSW/ACT were not statistically different when compared between 2002–2007 and 2008–2012 (incidence rate ratio = 1.13, 95% CI 0.76–1.69, *p* = 0.52).

There was a positive family history of neuromuscular disease in 15/107 (14.0%) of the cohort. Of this subset, 4/15 (26.7 %) had an older sibling <6 years of age and diagnosed with DMD only after the birth of the proband. Importantly, “theoretically preventable” cases were seen in 11/107 (10.3%) of the cohort. For these cases, the mother was already pregnant with the proband prior to or during diagnostic confirmation of disease for an older sibling in 5/11 (45.5%), 2/11 (18.2%) parents declined prenatal testing despite being a known carrier, 1/11 (9.1%) became aware of the family history of neuromuscular disease only after proband diagnosis, 1/11 (9.1%) had an incorrect carrier status assigned, and 1/11 (9.1%) did not understand carrier risk fully despite genetic counselling and cascade screening. For one proband the reason for “theoretical preventable” status could not be ascertained due to lack of clinical information. Although a downward trend in the incidence of “theoretically preventable” cases with time, from a peak of 3.6 per 100,000 in 2002–2004 to a nadir of 0.7 per 100,000 in 2008–2010 was observed (Table 2), the incidence rate ratio of “theoretically preventable” cases did not significantly change when compared between 2002–2007 and 2008–2012 (incidence rate ratio = 2.45, 95% CI 0.58–14.38, *p* = 0.18).

**Table 2:** The incidence of Duchenne muscular dystrophy and theoretically preventable cases in New South Wales and the Australian Capital Territory in four study intervals, 2002–2012.

Period	Total number of DMD cases	Number of theoretically preventable cases	Combined male live births NSW/ACT	Incidence of DMD per 100,000 MLB	Incidence of theoretically preventable cases per 100,000 MLB
2002–2004	30	5	139,605	21.5	3.6
2005–2007	29	3	142,413	20.4	2.1
2008–2010	30	1	152,743	19.6	0.7
2011–2012	18	2	107,138	16.8	1.9
**Total**	**107**	**11**	**541,899**	**19.7**	**2.0**

*DMD* Duchenne muscular dystrophy, *MLB* male live births.

Between 2002 and 2012, 141 potential 1^st^ degree carriers (mothers and sisters) were identified from 107 families through pedigree analysis. Of these, all mothers of probands were counselled on their carrier risk and offered carrier testing. Only 4/107 (3.7%) declined genetic testing. Where causative variants were not identified in the proband, two mothers were counselled on the potential for carrier risk and offered prenatal testing in subsequent pregnancies. In the 101 cases where carrier testing was accessed and completed, de novo variants were identified in 40/101 (39.6%) of the cohort, whilst in 61/101 (60.4%), carrier status in the mother was confirmed. A breakdown of the proportion of isolated cases over the four consecutive study intervals showed an increase from 0.29 in 2002–2004 and subsequent stabilisation thereafter (0.40–0.48) (Table 3).

**Table 3:** The proportion of isolated cases of Duchenne muscular dystrophy in New South Wales and the Australian Capital Territory in four study intervals, 2002–2012.

Period	Familial cases (maternal carrier status identified)	Isolated cases (de novo cases)	^a^Unknown inheritance	Percentage (%) of isolated cases
2002–2004	19	8	3	29
2005–2007	14	13	2	48
2008–2010	18	12	0	40
2011–2012	10	7	1	41
**Total**	**61**	**40**	**6**	**39**

*DMD* Duchenne muscular dystrophy, *MLB* male live births.

^a^Cases where the mothers declined genetic (carrier) testing or in which the causing variant was not confirmed in the proband.

Of all eligible 1^st^ degree relatives, 116/141 (82.3%) accessed carrier testing during the study period. Of 122 potential 2^nd^ degree relatives who were deemed at risk of being carriers, counselling of carrier risk was documented in 45/122 (37%). Data was limited as to the proportion of 2^nd^ degree relatives accessing carrier genetic screening.

Genetic confirmation of a DMD causing genetic variant was available in 104/107 (97%) of the cohort during the study interval. Of the population, 3/107 (2.8%) required muscle biopsies to confirm a diagnosis of DMD with two of these children, both presenting in 2002–2003, in the years following this study. Variants were identified through MLPA in the majority [78/104 (75%)] of cases, whilst in 16/104 (15.4%), DNA sequencing was required to confirm diagnosis. The remainder of the cohort were identified through multiplex PCR and/or muscle biopsy (10/104; 9.6%).

## Discussion

This epidemiological study is the first to investigate the incidence of DMD in an Australian state or territory since the start of the 21st century. Our findings add to the data from only three international studies that have evaluated the changing incidence of this disease over the last two decades [26].

Despite annual fluctuations, DMD disease incidence in NSW/ACT was comparable to the overall incidence of disease of 18.6 per 100,000 live male births, (equivalent to a risk of new DMD cases of 1 in 5377 live born males), for the period spanning 1960–1971 [22]. This contrasted with later studies encompassing the 25-year period from 1975 to 2001 that noted significant falls in disease incidence in NSW/ACT to 1 in 6022 live male births, attributed to the advent of genetic technologies and active cascade screening and counselling [16].

Our results align with the current global birth prevalence of DMD of 15.1–19.5 per 100,000 live male births seen in the most recent studies from Europe and USA, [19, 26] a fall from a peak global incidence of 22.6 cases per 100,000 live male births spanning 1950–1980 [32]. Thus, despite fluctuations in DMD incidence over the decades [16], the overall incidence of DMD remained essentially unchanged not only within the study period, but when compared over the last forty years, with an epidemiological plateau replicated across international jurisdictions [33, 34]. Subsequently, our results from both an Australian state and international perspective, indicate that despite the evolution of novel genetic technologies coupled with dedicated neurogenetic services that facilitate targeted cascade screening, the impact on DMD disease incidence from this approach has reached saturation over the last two decades [31].

The role of targeted variant analysis and cascade screening is aimed at restoring reproductive confidence for non-carriers and identifying “at risk” carriers, to inform of their reproductive options (prenatal testing, PGD, egg/ embryo donation) and effectively manage high disease risk to reduce long-term morbidity and mortality [35].

In contrast, the results from our study indicate no significant change in the incidence of theoretically preventable cases over time, mostly due to a combination of factors that are beyond the capability of the current clinical framework to effect, despite best efforts. These include delays in diagnosis of older siblings which prevented reproductive choices being possible for subsequent generations, an unknown family history prior to the proband’s diagnosis and parental choice to refuse prenatal/antenatal testing. Importantly however, two children in our cohort were born due to miscommunication and/or misunderstanding of maternal carrier risk, secondary to difficulties in ascertaining and/or communicating carrier risk based on CK alone despite a strong family history of neuromuscular disease (prior to the advent of molecular technologies to determine causative variants), resulting in lost opportunities for timely genetic counselling in this subgroup.

The incidence of “theoretically preventable” cases during the current study period was similar to the incidence of 2.4 “theoretically preventable” cases per 100,000 live male births seen between 1960–1971 in NSW/ACT [22]. In correlation with the stable disease incidence and number of “theoretically preventable” cases, the proportion of isolated cases remained comparable from 2005 onwards, emphasising the suggestion that current strategies aimed at changing the epidemiology of DMD in NSW/ACT were not impactful on a population level.

The aetiology of the stable disease incidence in DMD appears multifactorial. Familial cases in the majority, occur in the absence of any family history of DMD, and cascade screening and reproductive strategies are only activated when an index case is clinically recognised. Despite the evolution of technologies, the complexity of the variant spectrum within the dystrophin gene may render a minority of cases undetectable to cascade screening and impermeable to optimisation of reproductive choice [13].

The high mutation rate of the dystrophin gene, leads to a high number of sporadic cases that contributes to the unchanging disease incidence [36]. Gonadal mosaicism, where pathogenic variants arise early in spermatogenesis or oogenesis is also theorised to contribute to 10–15% of ‘sporadic’ cases,[37] making risk assessment for genetic counselling complex for families of apparently ‘de novo’ cases [38].

Subsequently, despite centralised laboratory services and integrated, long established neurogenetic models of care that aim for systematic genetic carrier identification, nearly 20% of 1^st^ degree relatives (mostly female siblings of affected males) did not access carrier screening despite high rates of counselling. For 2^nd^ degree relatives, access to appropriate counselling appeared to be a rate limiting step, with just over one third of potential carriers documented as receiving genetic counselling of risk. The decline in familial participation in cascade screening across the pedigree, replicates prior studies from Europe that have suggested barriers to effective cascade screening implementation including psychosocial, logistical and information i.e., family notification and health literacy related factors [18, 20].

Whilst current models of cascade screening facilitate reproductive choice in individual circumstances, novel models that shift the paradigm towards improving the timing and quality of genetic information are now imperative to facilitate reproductive choice based on best evidence for all families and break the plateau in disease incidence that is emphasised in this study. Although newborn screening (NBS) for DMD is emerging as a model to expedite timely diagnosis and facilitate early access to novel genomic therapeutic pathways for probands internationally,[39] its role as a strategy to influence disease incidence is limited to in theory, reducing the cases of ‘generational DMD’ that account for 12% of all children born with DMD [32]. In our cohort, 4 families with symptomatic siblings <6 years and may have benefited from NBS for DMD to facilitate early diagnosis and provide reproductive options, prior to the birth of the proband [24].

Prenatal testing for DMD is offered only to women who are at a known increased risk of DMD, and thus a significant proportion of women, represented by 92/107 (86%) of our cohort, giving birth to an affected boy, would have no recourse for this preventative strategy due to the absence of family history of disease [40]. Most couples known to be at risk of an affected male historically access prenatal testing to prevent the birth of a second affected boy [40].

Alternatively, reproductive carrier detection informs prospective parents of their risk of transmitting a genetic disorder such as DMD, facilitating individualised counselling and reproductive planning, to improve reproductive autonomy and informed decision making [41, 42]. This particularly improves reproductive opportunities for the majority of (familial) cases, with these options becoming particularly relevant as healthcare policy extends to publicly funded preimplantation genetic diagnosis (PGD) for single gene disorders such as DMD in Australia from November 2021 [43, 44]. Whilst expanded carrier testing for DMD is the next frontier of improving reproductive choice, barriers to implementation including technological limitations mean that dystrophin sequencing is not universally or routinely incorporated into commercial multiplex reproductive screening panels, currently [45, 46].

If extrapolated to our study, reproductive carrier screening may have facilitated informed reproductive choices for nearly 60% of couples in our cohort. The potential clinical utility and validity of reproductive carrier screening for DMD (within an expanded carrier screening panel of 22 monogenic inherited diseases) has been recognised in a study of 766 couples with no family history of genetic conditions. Here, DMD occurred with an incidence of 0.1% in at risk couples and was thus the sixth most common disease found in this cohort [47]. Ascertaining reproductive risk of disease guided access to PGD for DMD to facilitate reproductive risk management in this subgroup [47]. Accordingly, an Australian pilot reproductive genetic carrier screening project, involving a 1300 gene panel and inclusive of dystrophin variant analysis is underway to evaluate reproductive risk in 10,000 couples [30]. A multi-layered approach that involves reproductive carrier screening, prenatal/preimplantation genetic testing and newborn screening for DMD, alongside improved clinical detection of symptomatic cases may be considered as the most holistic and effective model of care to reduce the impact and change the epidemiology of this neurodegenerative and life-limiting condition [40].

Limitations of the study include the retrospective nature of the study design, precluding complete analysis of genetic carrier counselling and screening across the pedigree. The potential for under ascertainment of carrier screening uptake may be related to genetic testing being completed outside the jurisdiction of our neurogenetic centre and not being accurately documented within the proband record. The epidemiological definition of ‘theoretically preventable’ cases used in this study has inherent limitations including its inability to account for the nuances of individual reproductive options offered to families. This is especially pertinent in our cohort where a proportion of older siblings were diagnostically confirmed with DMD in close temporal relation to a pregnancy with a second affected child.

This study encompassed an era prior to next generation sequencing and at least one theoretically preventable case was determinant on not finding a causative variant in the proband and ascertaining carrier risk based on linkage. Incidence studies of the future may better reflect the evolution of genomic diagnostic capabilities that are available in the current landscape. This study was conducted within two states/territories of Australia and applicability to the national disease incidence of DMD requires further evaluation. Whilst ascertainment bias has been an inherent limitation of many previous epidemiological studies of DMD to date [26], the cross-referencing of two data registries including clinical and laboratory data sets in this study, has led to a high degree of ascertainment of cases in NSW/ACT.

This study has uniquely highlighted stability in DMD disease incidence and the limitations of cascade screening models of care to change epidemiological trends on a population level. These findings underpin the rationale for developing innovative strategies that enable ascertainment of reproductive risk at earlier stages in the reproductive journey for couples.

## Data Availability

The data central to this study is available from suitably qualified researchers through any reasonable requests and has been deposited at University of New South Wales Central data repository.
